# Liposomal Encapsulation in Food Systems: A Review of Formulation, Processing, and Applications

**DOI:** 10.1002/fsn3.70587

**Published:** 2025-08-04

**Authors:** Muhammad Abdul Rahim, Hamdy A. Zahran, Hafiza Madiha Jaffar, Saadia Ambreen, Mohamed Fawzy Ramadan, Fahad Al‐Asmari, Roberto Castro‐Muñoz, Eliasse Zongo

**Affiliations:** ^1^ Department of Food Science & Nutrition, Faculty of Medicine and Allied Health Sciences Times University Multan Pakistan; ^2^ Fats and Oils Department, Food Industries and Nutrition Research Institute National Research Centre Cairo Egypt; ^3^ University Institute of Diet and Nutritional Sciences, Faculty of Allied Health Sciences The University of Lahore Lahore Pakistan; ^4^ University Institute of Food Science and Technology Faculty of Allied Health Sciences, The University of Lahore Lahore Pakistan; ^5^ Department of Clinical Nutrition, Faculty of Applied Medical Sciences Umm Al‐Qura University Makkah Saudi Arabia; ^6^ Department of Food and Nutrition Sciences, College of Agricultural and Food Sciences King Faisal University Al‐Ahsa Saudi Arabia; ^7^ Department of Sanitary Engineering, Faculty of Civil and Environmental Engineering Gdansk University of Technology Gdansk Poland; ^8^ Laboratory of Research and Teaching in Animal Health and Biotechnology Universite Nazi Boni Bobo‐Dioulasso Burkina Faso

**Keywords:** food application, freeze‐drying, liposomal encapsulation, rheological properties, spray‐drying

## Abstract

Liposomal encapsulation is a crucial technique in food applications, offering protection and targeted delivery of bioactive compounds. This review focuses on the impact of thermodynamic factors on liposomal structures, specifically bilayers with large unilamellar vesicles and multilamellar vesicles. This study focuses on the chemical composition of liposomes, emphasizing the role of hydrophobic tails and sterols during phase transitions at various temperatures. This review also outlines the influence of liposome chemical composition and rheological characteristics, which determine encapsulation efficiency, mechanical stability, and potential functionality in various food matrices. The review highlights the use of conventional lipids to create stable liposomes via spray‐drying and fluidized bed coating for encapsulating solid food particles. The chemical composition of liposomes, particularly the type of phospholipids and inclusion of stabilizers, significantly affects their mechanical and rheological properties. These properties, including viscosity and viscoelastic behavior, influence liposome stability during processing and storage. High rigidity, imparted by saturated lipids or cholesterol, enhances structural integrity, while flexible membranes facilitate better encapsulation of large molecules or fragile bioactives. Furthermore, this work has addressed the importance of enzyme encapsulation and freeze‐drying techniques to preserve the enzyme activity in food processing, such as cheese production and fermentation. This study concludes by emphasizing the increasing demand for liposomal encapsulation in the food industry for preserving foods and delivering functional ingredients while also addressing key challenges and future research directions.

## Graphical abstract

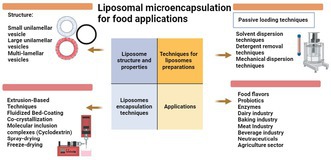

## 1 Introduction

**FIGURE 1:**
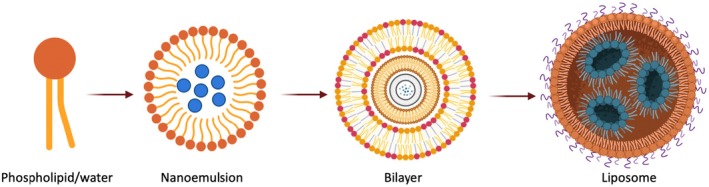
Phospholipid assembly process creates liposomes and bilayers.

The term “liposome” originates from the Greek words “lipo” (fat) and “soma” (structure), referring to a spherical shell structure with a liquid core surrounded by a phospholipid bilayer. Liposomes can encapsulate hydrophilic molecules in the aqueous phase and hydrophobic materials within the lipid membrane, making them versatile carriers (Shishir et al. 2019). The size of liposomes typically ranges from 50 to 500 nm in diameter, with various forms, including MLVs, LUVs, and small unilamellar vesicles (SUVs) (Has and Sunthar 2020). Liposomes with diameters ranging from 10 to 100 nm are considered optimal due to their favorable surface‐to‐volume ratio, which improves interaction with target sites and allows for efficient cellular uptake. Additionally, their small size promotes higher stability in colloidal suspensions and enhanced bioavailability of encapsulated compounds in food and pharmaceutical systems. Their structural, physical, and thermodynamic characteristics are similar to those of nanoliposomes. Lipids found in nature can self‐assemble into supramolecular aggregates such as micelles and bilayers. Lipid bilayers based on phospholipids aggregate into vesicles that Bangham named liposomes (Bangham et al. 1965) (Figure 1).

Since their discovery, liposomes have emerged as effective therapeutic nanoparticles, with several FDA‐approved liposomal medications for treating various illnesses and infections (Filipczak et al. 2020). Liposomes can encapsulate diverse substrates, ranging from tiny molecules to large biopolymers like DNA and RNA (Has and Sunthar 2020). By encapsulating these substances, it is possible to modify their biodistribution, prolong their circulation time, and impede their decomposition. Typically ranging from 80 to 150 nm in diameter, liposomes do not undergo renal clearance as their payload does. Alternatively, they may accumulate inactively in tumors and inflammatory areas through the Enhanced Permeability and Retention (EPR) effect (Maeda 2001).

While liposomes have been extensively studied for drug delivery (Filipczak et al. 2020), their potential in food science is increasingly recognized. Recent reviews have explored their application in food, digestion behavior, and absorption mechanisms (Liu, Kennedy, et al. 2020). This introduction critically analyzes previous and recent reviews on liposomal microencapsulation for food applications, addressing both conventional and novel techniques, such as supercritical fluid processes (Tsai and Rizvi 2016).

The formulation of liposomes plays a crucial role in determining their physicochemical properties, encapsulation efficiency, and overall suitability for food applications. The primary components of liposomes are phospholipids, whose hydrophilic head groups and hydrophobic tails self‐assemble into bilayer structures. The selection of lipid type (e.g., phosphatidylcholine, phosphatidylserine) influences the vesicle's stability and permeability. Cholesterol is often incorporated into formulations to modulate membrane fluidity, reduce permeability, and enhance stability during storage and processing. Stabilizers such as polyethylene glycol (PEG) or trehalose can be added to protect the liposome against aggregation, oxidation, and desiccation stress during freeze‐drying or spray‐drying (Zahran et al. 2023). The composition of the bilayer also impacts the compatibility of liposomes with specific bioactive compounds. Hydrophilic substances, such as ascorbic acid and certain peptides, are encapsulated within the aqueous core, while lipophilic compounds like curcumin, omega‐3 fatty acids, and vitamin D are incorporated into the lipid bilayer. The ability to carry both types of compounds makes liposomes a versatile delivery system in food matrices. Particle size and lamellarity (number of bilayers) are additional formulation parameters that affect liposome behavior. Smaller liposomes (e.g., SUVs) offer higher surface area and better cellular interaction, while larger multilamellar vesicles (MLVs) often provide sustained release. Surface charge, typically adjusted through the addition of charged lipids (e.g., phosphatidylglycerol), affects electrostatic stability and interaction with food components (Eugster and Luciani 2025). Bilayer rigidity, governed by lipid saturation and cholesterol content, influences resistance to mechanical stress during food processing. Together, these formulation factors significantly influence liposome stability, protect sensitive bioactives, and improve their bioavailability, thereby enhancing their applicability in various food products. This review aims to provide a comprehensive overview of liposomal microencapsulation in the food industry. It will cover liposome structures and properties, formulation methods, and the impact of processing techniques like spray‐drying and freeze‐drying on liposome stability and functionality (Lombardo and Kiselev 2022).

### 1.1 Liposome Structure

**FIGURE 2:**
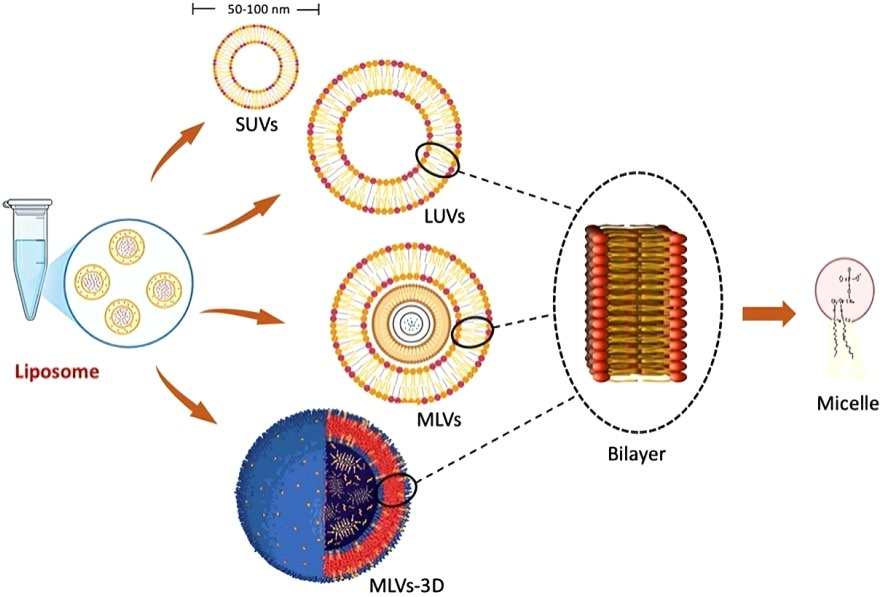
Three most prevalent forms of liposomes' structural attributes.

Liposomes exhibit diverse structures, varying in the number of concentric bilayers and overall size (Figure 2). These structural variations impact properties such as the stability and release rate of encapsulated materials. The primary types include small unilamellar vesicles (SUVs), LUVs, and MLVs.

### 1.2 Small Unilamellar Vesicles (SUVs)

SUVs consist of a single bilayer membrane, typically ranging from 20 to 50 nm in diameter. The phospholipid bilayer's thickness (~4 nm) and surface curvature limit the theoretical minimum size. SUV populations are generally uniform in size and well‐characterized (Liu et al. 2012). However, their single bilayer tends to be permeable, especially to water‐soluble substances, leading to lower entrapment efficiency due to the small entrapped volume. Thus, SUVs are better suited for lipophilic compounds (Romero‐Arrieta et al. 2020). SUVs, with diameters between 20 and 100 nm, are particularly effective in encapsulating lipophilic compounds such as curcumin, essential oils (e.g., limonene), fat‐soluble vitamins (e.g., vitamin D or E), beta‐carotene, and omega‐3 fatty acids, which have been successfully encapsulated in SUVs to provide specific context and improve clarity. Their single bilayer structure facilitates rapid diffusion and release, which is beneficial for enhancing the bioaccessibility of lipophilic nutrients in functional food applications. The high curvature of SUV membranes can lead to packing differentials and instability, with around 70% of phospholipids in the outer layer for liposomes ~25 nm in diameter (Khalili et al. 2020). Instability, like coalescence and fusion, increases at the phase transition temperature. Cholesterol is often added to improve stability by reducing or eliminating the transition. SUVs are generated by sonicating MLVs, extruding MLV solutions, or rapidly injecting phospholipid solutions (Sharma et al. 2018). However, these methods can be energy‐intensive and risk lipid peroxidation and hydrolysis. The stability of liposomes is influenced by the curvature of their bilayer membranes. Smaller vesicles such as SUVs exhibit higher membrane curvature, which increases packing stress and can reduce their thermodynamic stability. Conversely, larger vesicles like LUVs and MLVs possess lower curvature, leading to more stable bilayer arrangements. Cholesterol addition helps stabilize these membranes by reducing fluidity and preventing leakage of encapsulated materials (Lombardo and Kiselev 2022).

### 1.3 LUVs

LUVs are created by slowly introducing lipids and an ether solution into a phosphate buffer or fusing acid phospholipid tiny UVs with calcium. LUVs are very effective at encapsulating hydrophilic molecules because of their high phospholipid‐to‐aqueous phase ratio (Hanley et al. 2024). However, there are difficulties, such as mechanical instability and the possibility of retaining low molecular weight and water‐soluble solutes. Because LUVs are delicate, there are questions regarding how they would fare during food processing operations, which calls for more research into how resilient they are in such circumstances (Cavalcanti et al. 2022).

### 1.4 Multi‐Lamellar Vesicles (MLVs)

Multi‐lamellar vesicles (MLVs) are a liposome class characterized by several membranes and a broad variety of particle sizes (Lombardo and Kiselev 2022). Thin phospholipid films are made by rotary evaporating a chloroform solution containing phospholipid, cholesterol, and other hydrophobic substances. Lipid layer sheets split from the bulk when water and hydrophilic substances are added, creating liposomes with an alternating membrane and hydrophilic phase (Thabet et al. 2022). Unlike unilamellar vesicles, MLVs offer a more gradual and sustained material release because they are mechanically stable (van der Koog et al. 2022). Because the liposome contains a significant amount of phospholipid material, MLVs are efficient at ensnaring hydrophobic actives. However, because MLVs have a lower entrapment volume for a given size, they might not be the most effective way to transport hydrophilic actives. With unilamellar vesicles, the aqueous space ratio can be reached with the minimum lipids (Sharma et al. 2018).

### 1.5 Other Structures

Beyond SUVs, LUVs, and MLVs, other liposome structures exist, including intermediate unilamellar vesicles (IUVs) around 100 nm in diameter (Forooqi Motlaq et al. 2023), oligo‐lamellar liposomes, giant unilamellar vesicles, multivesicular vesicles, stable paucilamellar vesicles, helical liposomes, and cochleate cylinders (Shah et al. 2020). Liposomes produced by dehydration‐rehydration are known as dried‐reconstituted vesicles. Similarly, reverse‐phase evaporation and microfluidization yield reverse‐phase evaporation vesicles (REV) and micro‐emulsification liposomes (MEL), respectively (Farooque et al. 2021; Laffleur and Keckeis 2020).

### 1.6 Liposome Properties

Lipid vesicles, or liposomes, are aqueous solutions of amphiphilic substances, especially polar lipids, which tend to form bilayer structures (Walde and Ichikawa 2021). Liposomes are generally spherical and can include one or more layers of amphiphilic polymolecular membranes (Walde and Ichikawa 2021; Giuliano et al. 2021). When liposomes with a single bilayer membrane were first described, they were called “spherulites” because of their spherical shape (Eskandari et al. 2021). Currently, nevertheless, they are referred to as little (< 30 nm) or large (30–100 nm) unilamellar vesicles, or LUVs and SUVs, appropriately (Grimaldi et al. 2016). MLVs are liposomes with more than one bilayer membrane when all layers are concentric. If multiple randomly sized vesicles are encased within the interior of another vesicle, they are also known as multivesicular vesicles (MW) (Sreelaya and Bhattacharya 2023). The samples will appear hazy white as liposomes grow larger than roughly 300 nm because they will scatter light sufficiently for the unaided eye to notice (Londhe and Sharma 2022). However, further processing methods like sonication or extrusion, which are covered later, may produce smaller liposomes (Shah et al. 2020; Itrat et al. 2024). A solution with liposomes smaller than 300 nm appears clear or has a faint blue tint. As such, one of the most important characteristics of liposomes is the dispersion of their particle sizes both during and after creation (Shah et al. 2020).

Liposomes differ from surfactant micelles, which provide a hydrophobic environment because their interior makeup is primarily watery (Chevalier and Bolzinger 2019). This differentiation results from the orientation of polar lipids, which exposes the polar headgroups on the inner and outer surfaces of the membranes to their maximum extent relative to the corresponding inner and outer solvent phases. Consequently, the chemical composition of the aqueous solution inside liposomes is identical to that of the medium used to produce them (Has and Sunthar 2020). Adsorbent material can be incorporated into the bilayer's interior by solubilization, which is made possible by the non‐polar inner core of the membrane where the hydrophobic tails of polar lipids interact. Liposomes are watery inside, unlike surfactant micelles, which produce a hydrophobic environment (Wakaskar 2018). Polar lipids are oriented to maximize exposure to inner and outer solvent phases on the membrane's surfaces, resulting in the same chemical makeup as the medium in which they were first created. The hydrophobic tails of polar lipids interact in the non‐polar inner core of the membrane, allowing solubilization, the process of incorporating lipophilic material into the bilayer interior (Taylor et al. 2005).

Phospholipids make up the majority of the makeup of liposomes; however, other lipids, such as galactolipids, are also present. The majority of biological membranes are made up of two forms of phospholipids: phosphodimers and sphingolipids. Phospholipid‐rich lecithin, also known as phosphatidylcholine (PC), is primarily produced when two acyl hydrocarbon chains are ester‐linked to the glycerol position. Sphingolipids, essential for signal transmission, are produced when a ceramide, such as sphingomyelin, combines with a phospholipid (Panevska et al. 2019). Additional polar lipids utilized in liposomes consist of sterols such as sitosterol and cholesterol, kephalins, phosphatidyl‐ethanolamines, phosphatidyl‐serine, and phosphatidyl‐inositol. Systems containing phospholipids exhibit various phase behaviors, varying in composition and temperature from liquid‐crystalline to mesomorphic forms (Taylor et al. 2005).

One important property is the gel‐to‐liquid crystalline transition temperature (Tm), at which point the hydrocarbon chain melting causes the bilayer to lose most of its ordered packing structure. Higher phase transition temperatures are correlated with longer hydrocarbon chain lengths. Because of their asymmetric hydrocarbon chains, most natural phospholipids undergo transitions at different temperatures. The hydrocarbon chain's level of fatty acid unsaturation also affects the phase transition, with T decreasing as saturation rises (Pardauil et al. 2017). The phase transition temperature may also rise due to strong head group interactions. Complex liposomes can have bi‐ or multi‐component compositions. Cholesterol is frequently added to increase their in vivo *and* in vitro stability. This alters the interactions between the hydrocarbon chain and polar head groups, giving the membrane stiffness (Khalili et al. 2022).

Liposomes' functioning relies on their interaction with other chemicals, influenced mainly by their interfacial qualities, including rheological properties, hydrophobicity, and surface charge (Liu, Kennedy, et al. 2020). These characteristics depend on the bilayer's chemical makeup and external factors, including temperature, ionic strength, and the kind of solvent. Upon heating, bilayer structures can experience intricate phase changes, rendering them vulnerable to mechanical strains that could result in the disintegration and release of contained chemicals. The surface charge of liposomes influences lipid particles' electrostatic interactions with other charged substances (Zhao et al. 2022). Charged liposomal membranes may shield materials encapsulated inside the liposomes from solvent phase molecules by preventing equal‐charged compounds from entering the liposomes' core (Liu et al. 2018). Over a broad pH range, the zwitterionic head group of PC, a frequently used phospholipid, has a zero net charge. Electrophoretic mobilities, on the other hand, vary from +0.2 to −0.7μms1V−1 cm when different salts are present, and they drastically drop when the liposomes undergo a phase transition. The interactions between molecules in the membrane also affect the real surface charge of liposomes, in addition to the characteristics of the phospholipid molecule (Chen et al. 2024).

Protein interactions with bilayer structures are essential for metabolic processes, including the production of ATP. Proteins can be integrated into the membrane itself, or they can be incorporated into its exterior or interior surface (Almeida et al. 2017). Polar lipids, environmental factors, and molecular characteristics all influence how proteins are arranged in space within these membranes. Both intrinsic and extrinsic factors, such as phospholipid concentration, composition, and entrapped compound concentration, affect the characteristics and functions of liposomes (Nasr et al. 2020). Extrinsic factors include pH, ionic strength, and temperature. Liposomes' chemical and physical stability is influenced by these features. Polar lipids can undergo oxidation and hydrolysis, leading to chemical instability, whereas phase shifts or merging bilayer structures can destabilize physically (Van Tran et al. 2019). Under certain production and application conditions, food manufacturers must carefully select combinations of phospholipid and functional components to maximize capsule stability (Timilsena et al. 2020).

### 1.7 Methods of Preparation and Manufacturing

Reverse‐phase evaporation, ethanol dilution, thin‐film hydration, and other techniques can be used to efficiently create liposomes from formulations containing widely used lipids, such as cholesterol, phosphocholine (PC), phosphatidylglycerol (PG), and phosphatidylethanolamine (PE) (Lombardo and Kiselev 2022). A variety of compounds can be encapsulated by conventional lipids and create stable liposomes. To achieve stable liposome formulations, several physicochemical parameters must be optimized, including lipid type and concentration, pH, and ionic strength of the dispersing medium. The choice of aqueous buffer impacts vesicle stability and encapsulation efficiency. Incorporation of cholesterol enhances membrane rigidity and reduces permeability. Cryoprotectants such as trehalose or sucrose help maintain structural integrity during drying processes. These factors collectively influence liposome shelf‐life and functionality in food applications. There is a need for novel lipids with various shapes and functions to get past the current barriers in the liposome area and address therapeutic problems (Ahmed et al. 2019). Hydrophilic molecules from the aqueous phase may become lodged inside the liposome, whereas hydrophobic material may be incorporated into the lipid membrane during the liposome‐formation process (Has and Sunthar 2020). With liposomes, hydrophilic and hydrophobic substances can coexist in one structure (Ewert et al. 2021).

Proteins, lipids, carbs, vitamins, and minerals are only a few of the many chemical components that make up food structures. These ingredients' antioxidant, antibacterial, and flavor‐enhancing properties give them unique qualities. Grape seeds are recognized for their antioxidant and antibacterial qualities due to the presence of catechin and phenolic acids. By creating oil‐in‐water emulsions, these substances can improve microbiological safety and extend the shelf life of food products (Shoukat et al. 2024). Tan and McClements (2021) found that applying these chemicals via liposomes greatly enhances food quality. In food science, liposomes have garnered much interest as delivery mechanisms. Liposomes have specific benefits for encapsulating functional food components compared to other vesicles. According to Liu, Hou, et al. (2020), they offer a defense against environmental stressors, guarantee the preservation of encapsulated food ingredients, improve their functionality, and promote better absorption. Water‐soluble, oil‐soluble, or both can be encapsulated inside the architecture of liposomes (Janik et al. 2023). Liposomes have experienced minimal commercial usage in the food business despite their extensive use as carriers and excipients in the pharmaceutical and cosmetic industries. Liposomes are much smaller than other types of microencapsulation systems used in foods. Liposomes, due to their smaller size compared to other microencapsulation systems, offer a higher surface area‐to‐volume ratio, which enhances the bioavailability of encapsulated nutrients. Their nanoscale dimensions also facilitate better dispersion in food matrices, allowing uniform distribution. This helps maintain the sensory properties of food products without altering texture or appearance. Additionally, smaller liposomes are more efficient in crossing biological barriers, making them ideal for targeted nutrient delivery. These advantages support their potential in functional and fortified foods.

**FIGURE 3:**
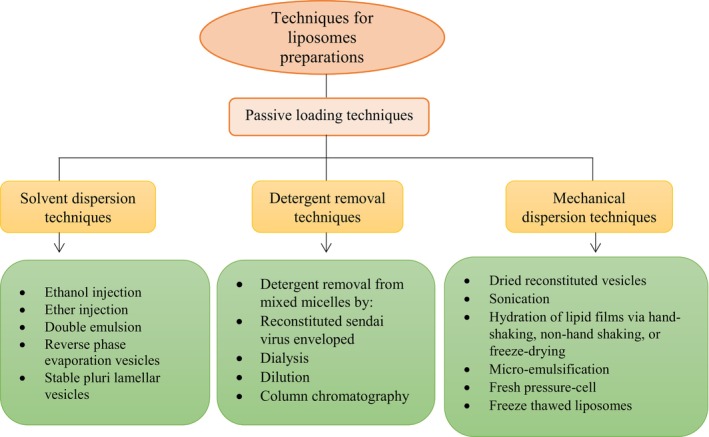
Distinct techniques for preparing liposomes.

The following factors determine the proper approach for liposome preparation (Figure 3): (1) the characteristics of the material to be entrapped, both physically and chemically, as well as the liposomal contents; (2) the kind of media in which the lipid vesicles are dispersed; (3) the material's effective concentration and potential toxicity; (4) additional procedures in the application or distribution of the vesicles; (5) the size, polydispersity, and shelf life of the vesicles that are optimal for the intended use; and (6) batch‐to‐batch reproducibility and the possibility of producing safe and efficient liposomal products on a large scale.

### 1.8 Liposome Handling

Proper handling of liposomes is crucial to maintain their structural integrity and functionality, especially when used in food applications. Specific factors, such as storage conditions, temperature, and interactions with other food components, can significantly affect liposome stability and encapsulation efficiency. Different liposome types serve distinct applications in the food and pharmaceutical industries. SUVs are ideal for delivering bioactives that require rapid absorption due to their small size and high surface area. LUVs are commonly used for sustained release applications and can encapsulate larger quantities of hydrophilic compounds. MLVs, with multiple concentric bilayers, are suitable for applications requiring prolonged protection and controlled release, such as encapsulating probiotics or antioxidants in dairy and beverage systems.

### 1.9 Storage Conditions

Liposomes are susceptible to degradation over time, which can lead to leakage of encapsulated materials and changes in particle size distribution. Therefore, appropriate storage conditions are essential to prolong their shelf life. Liposomes should be stored at temperatures that minimize lipid oxidation and hydrolysis. Generally, refrigeration (4°C) or freezing (−20°C) is recommended. However, repeated freeze–thaw cycles can damage liposomes, leading to aggregation and leakage (Sriwongsitanont and Ueno 2011). Lyophilization (freeze‐drying) is often employed to enhance long‐term storage stability. Exposure to oxygen can promote lipid oxidation, particularly in liposomes containing unsaturated fatty acids. Storage under an inert atmosphere (e.g., nitrogen or argon) can help minimize oxidation (Mozafari 2005). Lipids can be sensitive to light, which can accelerate degradation processes. Liposomes should be stored in dark or opaque containers to protect them from light exposure. The composition of the suspension medium can affect liposome stability. Buffers with appropriate pH and ionic strength should be used to maintain liposome integrity (Allen and Cullis 2013). Cryoprotectants like trehalose or glycerol are often added during freeze‐drying to protect liposomes from damage.

### 1.10 Reconstitution of Lyophilized Liposomes

Lyophilization (freeze‐drying) is a common method for preserving liposomes, but proper reconstitution is crucial to restore their original properties. The rehydration solution should be compatible with the encapsulated material and the liposome composition. Add the rehydration solution to the lyophilized liposomes gently to avoid disruption. Allow the liposomes to rehydrate for a sufficient period (e.g., 30–60 min) at room temperature or a refrigerated temperature. Gently mix the suspension to ensure uniform rehydration. Avoid vigorous shaking or sonication, which can damage the liposomes.

### 1.11 Interaction With Food Components

When incorporating liposomes into food products, it is important to consider their interactions with other food components. Extreme pH conditions can destabilize liposomes, leading to leakage or fusion. High salt concentrations can affect liposome stability and aggregation. Lipases and other enzymes present in food can degrade liposomes, releasing their contents. Enzyme inhibitors or encapsulation within a protective matrix can help mitigate this effect. Food processing operations such as homogenization, pasteurization, and mixing can subject liposomes to mechanical stress, potentially causing damage. Optimizing processing conditions is crucial to minimize liposome disruption.

### 1.12 Characterization of Liposomes

To ensure the quality and functionality of liposomes, it is important to characterize their properties before and after handling. Dynamic light scattering (DLS) is commonly used to measure liposome size and size distribution. This measures the percentage of the active compound encapsulated within the liposomes. Microscopy techniques such as transmission electron microscopy (TEM) or cryo‐electron microscopy (cryo‐EM) can visualize liposome structures. Monitoring changes in particle size, encapsulation efficiency, and leakage over time can assess liposome stability under different handling conditions. By carefully considering these handling factors and implementing appropriate storage and processing strategies, it is possible to maintain the integrity and functionality of liposomes for food applications, ensuring effective delivery of encapsulated bioactive compounds and improved product quality.

## 2 Recent Advances in the Technique

### 2.1 Encapsulation of Food Ingredients via Different Methods

Encapsulation is a crucial technique in the food industry to protect bioactive compounds, enhance their stability, control their release, and improve their bioavailability (Castro‐Muñoz et al. 2024; Ferreyra‐Suarez et al. 2024). While various encapsulation methods exist, liposomal encapsulation offers distinct advantages, particularly in addressing limitations associated with other techniques. This section provides a comparative overview, focusing on the benefits of liposomes over alternative encapsulation methods in the context of food applications. Emulsions are widely used for encapsulating hydrophobic compounds in aqueous systems. However, they often suffer from instability issues such as creaming, flocculation, and coalescence, leading to phase separation and reduced encapsulation efficiency (McClements 2015). Additionally, emulsions may not provide adequate protection against degradation factors such as oxidation or enzymatic activity. Microcapsules, formed through techniques like spray‐drying, extrusion, or coacervation, can provide a physical barrier to protect encapsulated ingredients. However, the high processing temperatures involved in spray‐drying can damage heat‐sensitive compounds. Extrusion may result in large particle sizes, affecting texture and mouthfeel in food products. Coacervation may require harsh chemical conditions that are not suitable for all food ingredients (Gharsallaoui et al. 2007). Hydrogels, made from polysaccharides or proteins, offer a biocompatible matrix for encapsulation. However, they may exhibit low encapsulation efficiency, poor mechanical strength, and limited control over release kinetics. Furthermore, hydrogels may be susceptible to degradation in the gastrointestinal tract, leading to premature release of encapsulated compounds (Anal and Singh 2007).

### 2.2 Advantages of Liposomal Encapsulation

Liposomes offer several advantages over traditional encapsulation methods, making them a promising delivery system for food ingredients:

- *Enhanced encapsulation efficiency*: Liposomes can encapsulate both hydrophilic and hydrophobic compounds within their aqueous core and lipid bilayer, respectively, offering higher encapsulation efficiency compared to methods that are limited to one type of compound (Anandharamakrishnan 2022 ).
- *Improved stability*: The phospholipid bilayer of liposomes provides a protective barrier against degradation factors such as oxidation, enzymatic activity, and pH changes, enhancing the stability of encapsulated ingredients during processing, storage, and digestion (Mozafari 2005).
- *Controlled release*: Liposomes can be designed to release their contents in a controlled manner, either through passive diffusion, triggered release, or targeted delivery to specific sites in the gastrointestinal tract. This allows for improved bioavailability and targeted delivery of bioactive compounds (Allen and Cullis 2013).
- *Biocompatibility and biodegradability*: Liposomes are composed of naturally occurring phospholipids, making them biocompatible and biodegradable. This reduces concerns about toxicity and accumulation in the body, making them suitable for food applications.
- *Versatility*: Liposomes can be tailored to specific applications by modifying their lipid composition, size, surface charge, and targeting ligands. This allows for the development of customized delivery systems for a wide range of food ingredients (Mallick and Choi 2014).

## 3 Specific Examples

- *Omega‐3 fatty acids*: Liposomes can protect omega‐3 fatty acids from oxidation, a major challenge in incorporating these compounds into food products. Traditional emulsions often fail to provide adequate protection, leading to rancidity and loss of nutritional value (Shahidi and Zhong 2005).
- *Enzymes*: Liposomes can encapsulate enzymes to protect them from denaturation or inactivation during food processing. This is particularly useful in applications such as cheese making or baking, where enzymes play a crucial role in product quality (Gandhi 2009).
- *Vitamins and antioxidants*: Liposomes can enhance the stability and bioavailability of vitamins and antioxidants, which are often sensitive to degradation during processing and storage. This can lead to improved nutritional value and health benefits of food products (Uttreja et al. 2022).

Liposomal encapsulation offers significant advantages over traditional encapsulation methods for food ingredients, including enhanced encapsulation efficiency, improved stability, controlled release, biocompatibility, and versatility. By addressing the limitations of other techniques, liposomes provide a promising approach to protect, deliver, and enhance the functionality of bioactive compounds in food products. Further research and development are needed to optimize liposome formulations and processing conditions for specific food applications. Liposomes are typically composed of phospholipids such as phosphatidylcholine and cholesterol, forming bilayered vesicles that can encapsulate both hydrophilic and lipophilic substances. The formulation of liposomes is influenced by several factors, including the type and concentration of lipid components, the method of preparation, and environmental conditions (e.g., temperature and pH). Cholesterol is commonly incorporated to improve membrane stability and reduce permeability. Additives such as polyethylene glycol (PEG) can be used to enhance circulation time in vivo. Table 1 summarizes common ingredients and their specific functions in food‐grade liposome preparations.

**TABLE 1:** Liposomal spray‐drying in food applications.

Liposome composition	Encapsulated compound	Spray‐drying parameters	Cryoprotectant/Stabilizer	Encapsulation efficiency (%)	Particle size (nm)	Stability/Retention	Food application	References
Phosphatidylcholine, cholesterol	Vitamin C	140°C inlet, 80°C outlet	Trehalose	85	180	90% after 3 months	Juice fortification	Secolin et al. (2017)
Soy lecithin	Curcumin	150°C inlet, 90°C outlet	Maltodextrin	78	210	88% after 6 months	Functional dairy	Ang et al. (2024)
Egg phospholipid	Omega‐3 fatty acids	130°C inlet, 75°C outlet	Sucrose	82	170	85% after 4 months	Baked goods	Chougule et al. (2008)
Gelatin	MCT oil (lipophilic)	150°C inlet, 90°C outlet	Tween 80	87	280	90% after 3 months	Functional dairy	Wang et al. (2017)
Liposomes + Trehalose	Paclitaxel (PTX)	60°C inlet, 50°C outlet	Trehalose	96.74	520	83 after 4 months	Baked goods	Dattani et al. (2025)
Soya lecithin, cholesterol	Insulin	150°C inlet, 90°C outlet	Maltodextrin	43.60	1000	75 after 3 months	Functional food	Bi et al. (2008)
DPPC: Cholestrol (7:3) liposome+ sucrose + l‐leucine	Dapsone	150°C inlet, 90°C outlet	Sucrose	97.9	170	90% after 3 months	Topical application	Deshkar et al. (2018)

### 3.1 Extrusion‐Based Techniques

All unstable flavors have been encapsulated in glassy carbohydrate matrices utilizing extrusion microencapsulation. The key benefit of this method is the significantly increased shelf life given to flavor chemicals, which are often prone to oxidation. Although some of these techniques, such as fluid bed spraying, might potentially use glassy carbohydrates as shell elements, extrusion is still the best option (Zasypkin and Porzio 2004). This process includes depositing particles of a polymer's aqueous phase into a solution starting to gel. The commonly utilized copolymer with calcium chloride gelation bath solution is sodium alginate (0.6%–3%). A dropper, needle, vibrating needle sprinkling shower head, jet cutter, or vibrating plate can be used as a dropping tool. This technique is frequently used to isolate volatile ingredients and unpredictable flavors. Co‐extrusion is used for concentrated and slippery cores or polymeric integration and particle manufacturing, resulting in the structure of matrix crystallite as compared to traditional formation, which is typically used for particle falsifications by forcing a blend of core and encapsulates through the nozzle (Schäfer et al. 2018). The founder produces small capsules with a multilayer structure as a core and encapsulates flow from a similar outlet at a specific rate, while the stream rate of the encapsulates and steel and the vibrating grid connected shell depth (Petraitytė and Šipailienė 2019). Alginate is negatively charged over various pH values and can create a gel by binding with metal ions.

With increased alginate and cations, such as calcium chloride solution, enough bridge formation occurs and drug entrapment is increased, making Ca^2+^ the most commonly employed cation (Villanueva‐Bermejo et al. 2017). The extrusion capsules can be dried by spray‐drying or cold pressing to improve physical stability. Freeze‐drying results in permeability, which affects encapsulation effectiveness and reduces core protection. Alginate pearls also have a higher propensity for the fast release of active components because of their reduced percentage (2% to 4% alginate hydrogel of increased viscosity), which produces a low‐density gel system during digestion or in an aqueous phase (Fernandes et al. 2013). Alginate is mixed with positively charged bioplastics like glucosamine to solve the problem of quick disconnect. Moreover, it was unearthed that the use of dietary natural fibers like microbeads isolated from prebiotic fiber and rice, coupled with alginate before being bridged with calcium chloride, substantially enhances the enclosing efficiency and enables the best release of additives like caffeine (Belščak‐Cvitanović et al. 2015). Due to the charge transfer and complexing effects of these beads, Sarıyer et al. (2020) used mixtures of carrageenan and alginate, and potassium chloride and calcium chloride salts to increase encapsulating efficiency and sustained release.

### 3.2 Fluidized Bed‐Coating

This approach applies to fluidizing solid particles, an adapted version of the spray thermal evaporation (Frakolaki et al. 2020). The drug industry is where it is mainly used. In this method, porous solid material is absorbed to encapsulate the solid or liquid core materials. It includes three steps: floating the components in the air, spraying molten phase to cover the hanging suspended solids, and final chilling or vaporizing solvents to firm the shell. Electrostatic, hydrophobic, and hydrophilic contacts, interfacial tension, and stickiness are all essential parts of the envelop solution that hold the core material to the encapsulates (Benelli and Oliveira 2019). These three steps will be repeated until the necessary inner surface is reached. This method has been used to minimize the amount of ascorbic acid using polymethyl acrylate and ethyl cellulose. Ascorbic acid was hydrated and released by Knezevic et al. (1998) utilizing polymethacrylate coating and hydrophobic coating materials as the highest. *Rosmarinus vulgaris* extract, including a range of bioactive polyphenols, including carnosol, means different, canonic, and caffeoylquinic acids, was encapsulated by Benelli and Oliveira (2019) utilizing a fluidized bed spraying coating with gum Arabic and whey protein concentrate as encapsulation solutions. When drying air temperatures reached 70°C, the authors reported more than 75% encapsulation rates for carnosic acid, rosmarinic acid, and carnosol; for caffeic acid, this was made achievable using a less viscous whey protein concentrate solution.

Moreover, gum Arabic, a viscous fluid solution, exhibited lower encapsulation effectiveness (57%) than other solutions due to poor caffeic acid trapping. Sun et al. (2013) discovered a 95% encapsulation efficiency when encapsulating l‐methanol with a gelatin emulsion as an encapsulated solution and a decreased encapsulation efficiency when using a collagen solution with a high viscosity. According to various scientists, the ideal interfacial tension of encapsulates should be between 32 and 49 mN/m since this allows for a perfect architecture, homogeneous plate thickness, and increased encapsulation effectiveness (Yun et al. 2021). The most popular fluidizing and washing medium is air; warm air directly affects crystallites' quality. For instance, high temperatures can lead to pore spaces, fissures, and irregular wall thickness and coating because particles are dried before coming into contact with the substrate of the core material (Havaić et al. 2018). Increased coating cycles, correct coating solution flow and air velocity, temperature, ionized plasma pressure, core‐to‐encapsulate ratio, and the selection of coating materials with desired traits such as lower density, creating the possibility of poor resistance, etc., all help to produce more beneficial microstructures (Seyedin et al. 2018; Pitigraisorn et al. 2017). Semyonov et al. (2012) used molten wax and polyvinyl alcohol solution (7% w/w) to encapsulate *Streptococcus piracies* and found that putty particles had a greater chance of survival at an input temperature of 45°C and an output temperature of 25°C. The researchers also pointed out that the ethyl cellulose coating reduced survival because the wall started to break. This method's main disadvantages continue to be a hard and drawn‐out process, unregulated particle aggregation brought on by cement slurry, and individual droplets between particles (Martín et al. 2015).

### 3.3 Co‐Crystallization

The sugar's crystalline phase is charged during the membrane fabrication, called co‐crystallization, from a flawless to an irregular flocculated crystal, creating a porous material that can hold a second active form (Castro‐Muñoz, Correa‐Delgado, et al. 2022). During this method, super‐saturated sucrose liquid is heated above 120°C to a Brix level of more than 95, and at this point, continuous swirling results in uncontrolled initiation and crystallization. A considerable quantity of heat is produced as the syrup reaches the point where conversion and crystallization start (Fang and Bhandari 2010). Agitation is continued to promote and enlarge the transition until the aggregates are released from the vessel later. If the second component, such as a taste component, is added right before the mixing, it will be integrated into the crystal globules in the blank land. The entrapment totally shields the flavors from the atmosphere outside (Augustin and Hemar 2009). Improved solubility, water sorption, uniformity, dissolution rate, dehydration, attenuator, stability, and encapsulated materials are the key benefits of co‐crystallization. Additional advantages include the capability to directly tablet the items due to their flocculated structure and the ability to convert the core materials from a liquid state to a dry powder without extra drying (Siddiqui et al. 2024). These characteristics substantially benefit the drug companies and candy industries (Desai and Jin Park 2005). Precise control of the temperature balance and the rate of initiation and crystallization during several stages of the method is vitally essential for producing good co‐crystallized aggregates, even though this process is simple and requires no specialized technology. The co‐crystals may require extra crushing and filtering to create grains with a certain particle size range. Yerba mate ( *Ilex paraguariensis* ) extract and honey orange rind oil were successfully encapsulated using this technique, which is especially helpful when high fundamental ratios are unnecessary (Deladino et al. 2008). Liposomes are dispersion particles that enclose a watery space within a membranous membrane made of lipid molecules (including lecithin or lecithin + cholesterol). An electrophilic interaction between phosphatidyl and moisture content is the primary process for creating liposomes and nanoliposomes. Bioactive compounds encapsulated in liposomes can be protected from stomach digestion and exhibit high absorption levels through the gastrointestinal tract, increasing their biocompatibility and availability (Garza‐Cadena et al. 2023). Liposomes have granules ranging from 30 nm to a few micrometers. There are a variety of procedures to produce liposomes, and research (Mozafari 2005) details the most widely used manufacturing methods. The typical production process includes creating a thin film by condensing lecithin, cholesterol, and other hydrophilic substances in a dichloromethane solution.

When a substantial quantity of heat or mechanical energy is applied after adding an aqueous layer and aqueous substance, multilayer sheets of the hydrophobic components break from the majority of liposomes (Reza Mozafari et al. 2008). The encapsulation at the industrial level is carried out using five different liposome‐making techniques, including thin film evaporation, ultrasound‐assisted, reverse phase evaporation, melting, and super cold; this latter one has been the subject of a comparative investigation (Fan et al. 2007). Another commonly used technique for liposome formation is high‐pressure homogenization or microfluidization (Thompson and Singh 2006). Thin film evaporation can create MLVs, reverse phase evaporation can produce bigger MLVs, and sonication or extrusion can generate smaller unilamellar cells. Extremely cold MLVs are generated due to the cooling process (Maestrelli et al. 2006). Most often used to encapsulate enzymes and antioxidants, liposome is primarily studied and utilized as complex medicinal medication carriers (Reza Mozafari et al. 2008). Serious issues include physical and chemical stability during low encapsulation production, storage, water‐soluble active agent loss during storage, use of acids and surfactants that are not meal, small‐scale production, and the cost of raw materials (Zuidam and Shimoni 2009). Due to recent advances in liposome technologies, including the heat source and the microfluidization method, liposomes can now be mainly produced without solvents and with reasonably high encapsulation efficiencies (Desai and Jin Park 2005). A great future for liposome encapsulation of food ingredients would depend on the invention of low‐cost thermal evaporation and a dry liposome composition that quickly reassembles without aggregation and leaking upon rehydration (Reza Mozafari et al. 2008).

### 3.4 Molecular Inclusion Complexes (Cyclodextrin)

Inclusion complex formation is a form of chemical personality in which a macromolecule forms a cage‐like structure to enclose one or more different kinds of molecules. In light of this, an integral membrane is considered an efficient encapsulation technique in which hydrophobicity is the primary driving force (Zhu et al. 2019). There is an ongoing discussion of the many ways in which cyclodextrin inclusion complexes (CD‐IC) (Kfoury et al. 2016). However, kneading (pasta), drying, and freeze‐drying have been the most popular methods in recent years. Furthermore, a two‐step process consisting of IC generation and drying has been developed after the effectiveness of these techniques was compared. Typically, cyclodextrin inclusion complex formation has obtained trapping efficiency (EF) between 70% and 80% (Kfoury et al. 2018; Rodríguez‐López et al. 2020). Encapsulation in cyclodextrins may increase the bioavailability of limited nutraceuticals and improve membrane penetration and dissolution rate (Hernández‐Pinto et al. 2024).

Additionally, CDs can serve as taste transporters by preserving flavors from oxidation and derivatives brought on by heat and light. In addition, cyclodextrins can prolong the shelf life of food items and cover up or lessen unpleasant taste and smell (Mourtzinos et al. 2007). The usage of cyclodextrins has advanced recently, particularly in encapsulating medicinal or nutraceutical compounds, such as polyphenols.

Cyclodextrins are heterocyclic compounds with a shortened conic shape, making them “ready encapsulating (wall) material.” d‐glucopyranoide units joined together by 1,4‐glycosidic bonds create them (Jansook et al. 2018). These molecules' external walls are hydrophilic and water‐permeable due to the locations of the secondary and primary hydroxyl groups. On the other hand, because of the glycoside bonds' position, the cyclodextrins' internal cavity is relatively hydrophobic (Crini et al. 2018). As a result, cyclodextrins, as compounds from extracts, can form inclusion complexes with a broad variety of hydrophobic molecules. Cyclodextrins (CDs) are macrocyclic oligosugars created by the enzymatic alteration of maize, and by absorbing small molecules and polymers into their tiny holes, they can generate inclusion compounds with non‐covalent bonds (Musuc 2024). The first and the last glucose molecules are linked to create a closed circular chain with 1–4 links just after starch dissociation mediated by the set of enzymes known as cyclodextrin‐glycosyltransferases. CDs' importance lies in these macrocyclic guests' ability to form complexes with other, often smaller molecules (known as guests) that can enter their cavities. Cyclodextrins can work together to form inclusion complexes with a wide variety of chemical and inorganic substances, penetrating the relatively hydrophobic cavity of the host partially or entirely. The molecular arrangement inside the CD structure is not hydrophobic, but it is significantly less hydrophilic than the aqueous phase, allowing it to carry hydrophobic compounds. Contrarily, the macrocyclic surface is hydrophilic enough even to confer the cyclodextrins' distinctive solubility in water (Kalogeropoulos et al. 2012).

### 3.5 Spray‐Drying of Liposomes

**FIGURE 4:**
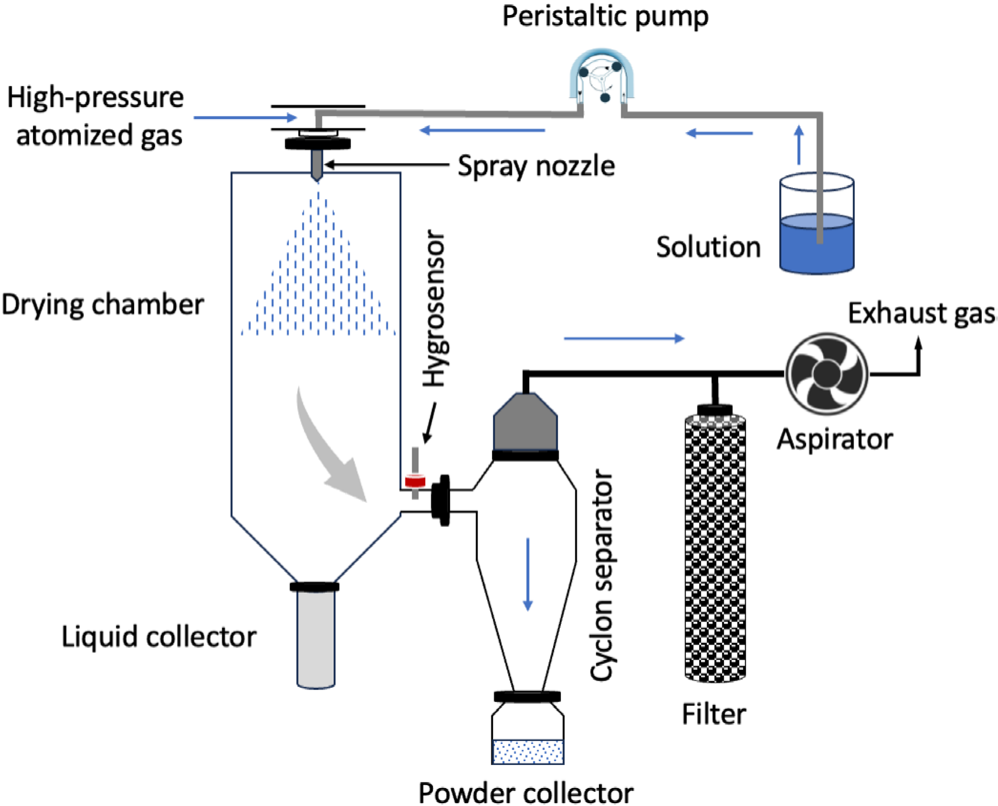
Schematic diagram of a spray‐dryer.

Spray‐drying is a widely used technique for producing dry powders from liquid feedstocks (Figure 4), offering advantages such as continuous operation, scalability, and relatively low cost (Gharsallaoui et al. 2007). In the context of liposomes, spray‐drying is employed to convert aqueous liposome dispersions into dry powders, enhancing their storage stability and ease of handling. However, the spray‐drying process can subject liposomes to various stresses, including heat, shear forces, and dehydration, which can compromise their structural integrity and encapsulation efficiency.

### 3.6 Effect of Spray‐Drying Conditions on Liposomes

The spray‐drying process involves several parameters that can affect liposome properties, including:

- *Inlet temperature*: High inlet temperatures can lead to lipid phase transitions, vesicle fusion, and leakage of encapsulated materials (Santos et al. 2018; Rahim, Imran, et al. 2024). However, lower inlet temperatures may result in incomplete drying and powder caking. Optimizing the inlet temperature is crucial to balance drying efficiency and liposome integrity (Rahim, Imran, Khan, Ahmad, et al. 2022).
- *Feed rate*: The feed rate affects the particle size and moisture content of the dried powder. Higher feed rates may result in larger particles with higher moisture content, potentially leading to instability issues (Vehring 2008).
- *Atomization pressure*: The atomization pressure influences the droplet size and drying rate. Higher pressures produce smaller droplets with faster drying rates, but can also generate greater shear forces that damage liposomes (Vehring 2008).
- *Carrier material*: The choice of carrier material can significantly impact liposome stability during spray‐drying. Sugars such as trehalose, sucrose, and lactose are commonly used as cryoprotectants to protect liposomes from dehydration‐induced damage (Ishwarya et al. 2022). Polymers like maltodextrin and modified starch can also be used to enhance powder flowability and dispersibility.

### 3.7 Challenges and Solutions

Spray‐drying can induce liposome aggregation and fusion, leading to increased particle size and reduced encapsulation efficiency. Incorporating cryoprotectants, such as trehalose, can help maintain liposome integrity by forming a glassy matrix that prevents aggregation and fusion during drying (Rahim, Imran, Khan, Haseeb Ahmad, et al. 2022). High temperatures and shear forces can cause leakage of encapsulated materials from liposomes. Optimizing spray‐drying parameters such as inlet temperature, feed rate, and atomization pressure can minimize leakage. Additionally, using lipids with higher transition temperatures can improve liposome stability at elevated temperatures (Santos et al. 2018). Spray‐dried liposome powders may exhibit poor flowability and dispersibility, making them difficult to handle and incorporate into food products. Adding flow aids like silicon dioxide or using carrier materials with good flow properties can improve powder flowability and dispersibility. Microencapsulation of liposomes within a larger matrix can also enhance powder handling characteristics (Fang and Bhandari 2010). Several studies have investigated the effect of spray‐drying on liposome properties. For example, Costa et al. (2025)) found that spray‐drying liposomes containing quercetin at an inlet temperature of 120°C resulted in significant degradation of quercetin and reduced encapsulation efficiency. However, incorporating trehalose as a cryoprotectant improved quercetin stability and encapsulation efficiency. Similarly, Ishwarya et al. (2022) demonstrated that spray‐drying liposomes containing insulin with trehalose resulted in a stable powder with good insulin activity. Spray‐drying is a viable technique for producing dry liposome powders for food applications. However, careful optimization of spray‐drying parameters and selection of appropriate carrier materials are crucial to maintain liposome integrity and encapsulation efficiency. Future research should focus on developing novel spray‐drying strategies that minimize liposome damage and enhance powder properties for specific food applications.

### 3.8 Spray‐Drying in Nutraceutical Applications)

Spray‐drying plays a crucial role in preserving the stability and efficacy of nutraceuticals, particularly in the encapsulation of vitamins. Vitamins, essential micro‐nutrients with biochemical functions, are susceptible to denaturation and damage during food processing. Techniques such as micro‐ and nanoencapsulation, including spray‐drying, are employed to preserve vitamins effectively (Pattnaik et al. 2021).

A study comparing three encapsulation methods, freeze‐drying, spray‐drying, and drum drying, discovered that freeze‐drying showed the least amount of β‐carotene (vitamin A) degradation (Desobry et al. 1997). Vitamin C, known for its antioxidant properties, was effectively encapsulated using materials such as methacrylate copolymers, chitosan, and pea protein, with varying encapsulation efficiencies and release characteristics (Hashim et al. 2025). Essential dietary components are minerals, such as iron, iodine, potassium, sodium, phosphorus, magnesium, and calcium. Spray‐drying has been used successfully to enrich meals with these minerals. For example, calcium microparticles were enclosed using neutral polymethacrylate and cellulose derivatives. Proper encapsulation reduces reactivity with other substances and ensures optimum release for dietary absorption, which is necessary for the bioavailability of certain minerals (Kurek et al. 2024).

Spray‐drying has also found applications in preserving the color and antioxidants in food, particularly in encapsulating pigments like lycopene and anthocyanins. The application of encapsulating flavors is well‐established, and common encapsulating materials include gum arabic, maltodextrin, and modified starch. To improve stability and preserve flavors, the encapsulation of essential oils from several sources, including citrus peel, cardamom, and oregano, has been investigated (Kaderides et al. 2024; Rahim, Ayub, et al. 2023). Spray‐drying has been used to encapsulate oils high in omega‐3 fatty acids, such as avocado and fish oil, in the context of essential fatty acids. These encapsulation methods aim to prevent oxidation and enhance stability, ensuring the delivery of health benefits associated with essential fatty acids (Rahim, Yasmin, et al. 2022; Abozed et al. 2021). Overall, spray‐drying emerges as a versatile technique in the nutraceutical and food industry (Table 1), offering effective encapsulation solutions for a wide range of bioactive compounds, vitamins, minerals, pigments, flavors, and essential fatty acids. Ongoing research continues to explore novel applications and optimize the process for enhanced preservation and delivery of bioactive ingredients in various food and nutraceutical products (Venugopalan et al. 2021).

## 4 Freeze‐Drying (Lyophilization) of Liposomes

Freeze‐drying, also known as lyophilization, is a dehydration process that involves freezing the material and then reducing the surrounding pressure to allow the frozen water to sublimate directly from the solid phase to the gas phase. Freeze‐drying is widely used to preserve labile materials, including liposomes, by removing water and reducing degradation reactions (Franks 1998).

### 4.1 Effect of Freeze‐Drying Conditions on Liposomes

The freeze‐drying process involves several stages that can affect liposome properties:

- *Freezing rate*: The rate of freezing can influence ice crystal formation and liposome damage. Slow freezing may result in the formation of large ice crystals that disrupt liposome structure, while rapid freezing can lead to smaller, more uniform ice crystals with less damage (van Winden et al. 1997).
- *Annealing*: Annealing involves holding the frozen material at a temperature above its glass transition temperature to promote ice crystal growth and reduce amorphous regions. This can improve liposome stability during drying (Franks 1998).
- *Drying temperature and pressure*: The drying temperature and pressure affect the sublimation rate and residual moisture content. Higher temperatures and lower pressures can accelerate drying but may also increase liposome damage (Franks 1998).
- *Cryoprotectants*: Similar to spray‐drying, the addition of cryoprotectants is crucial to protect liposomes during freeze‐drying. Sugars like trehalose, sucrose, and glucose are commonly used to maintain liposome structure and prevent aggregation (Ishwarya et al. 2022).

### 4.2 Challenges and Solutions

Freeze‐drying can induce liposome fusion and aggregation due to dehydration stresses. Using cryoprotectants like trehalose can prevent liposome fusion and aggregation by forming a glassy matrix that maintains liposome structure (Ishwarya et al. 2022). Freeze‐drying can cause leakage of encapsulated materials due to membrane disruption. Optimizing freezing and drying parameters can minimize leakage. Additionally, using lipids with higher transition temperatures can improve liposome stability during freeze‐drying. Freeze‐dried liposomes may be difficult to reconstitute, forming aggregates or exhibiting reduced encapsulation efficiency. Adding lyoprotectants like glycerol or mannitol can improve liposome reconstitution by promoting rapid and uniform hydration (van Winden et al. 1997). Several studies have investigated the effect of freeze‐drying on liposome properties. For example, Ishwarya et al. (2022) found that freeze‐drying liposomes containing insulin with trehalose resulted in a stable powder with good insulin activity after reconstitution. van Winden et al.(1997) demonstrated that rapid freezing and annealing improved liposome stability during freeze‐drying. Freeze‐drying is an effective technique for preserving liposomes for food applications. However, careful optimization of freezing and drying parameters, along with the use of appropriate cryoprotectants, is crucial to maintaining liposome integrity and encapsulation efficiency. Future research should focus on developing novel freeze‐drying strategies that minimize liposome damage and enhance reconstitution properties for specific food applications.

The versatility of liposomes stems from their ability to be tailored for specific applications through adjustments to lipid composition, size, surface charge, and the incorporation of targeting ligands. The following Table 2 highlights a selection of liposome formulations, detailing their lipid composition, size, encapsulation efficiency, and stability characteristics, as well as their application in the food industry. These examples illustrate the potential of liposomes for targeted delivery of bioactive compounds, enzymes, and other food ingredients, contributing to enhanced product quality, stability, and nutritional value.

**TABLE 2:** Liposome formulations and properties.

Formulation	Lipid composition	Size (nm)	Encapsulation efficiency (%)	Stability	Application	References
Liposome A	PC/Cholesterol (2:1 M ratio)	100–200	75–85	Stable at 4°C for 3 months, protected from light and oxygen	Omega‐3 fatty acid delivery	Shahidi and Zhong (2005)
Liposome B	Soy lecithin/DPPG (9:1 M ratio)	80–150	65–75	Stable at −20°C with 10% Trehalose, Lyophilized	Enzyme (e.g., Lysozyme)	Gandhi (2009)
Liposome C	DMPC/Cholesterol/DSPE‐PEG (55:40:5)	70–120	80–95	Stable at 25°C for 1 month, protected from enzymatic activity	Vitamin C/Antioxidant delivery	Uttreja (2022)
Liposome D	Egg PC/Cholesterol (7:3 M ratio)	120–250	70–80	Stable at 4°C for 2 months	Probiotic encapsulation	Anal and Singh (2007)
Liposome E	Phosphatidylcholine (PC)	50–300	60–90	Depends on storage condition.	General food ingredient delivery	Shishir et al. (2019)

## 5 Principle and Process of Freeze‐Drying

There are three states of water: solid, liquid, and gas. The change from a solid to a vapor (sublimation), evaporation, or fusion is depicted in the phases. The three water phases coexist at the triple point of 0.01°C and 0.612 kPa (Bhatta et al. 2020). The critical point is located at 22,060 kPa and 374°C. Sublimation occurs at temperatures below 0.01°C and water vapor pressures below 0.612 kPa, which is how freeze‐drying works (Yao et al. 2023; Bhatta et al. 2020). A product that is to be freeze‐dried travels from point A to point B, freezing due to temperature drops, a drop in water vapor pressure below the triple point, and applying heat to turn ice into vapor (Ward and Matejtschuk 2020).

The freeze‐drying (FD) method consists of three steps: primary drying, secondary drying, and freezing. The first stage of separation is freezing, which solidifies food ingredients. The pace of freezing influences ice crystal formation and size; larger crystals are formed at a slower rate and vice versa. Larger ice crystals accelerate primary drying because they sublimate more readily (Pardeshi et al. 2023). Primary drying entails creating a vacuum and raising the shelf temperature to initiate sublimation. The collapse temperature (Tc) at which the product is most likely to lose its macroscopic structure should be 2°C–3°C below this value (Feng et al. 2023). Tc can be determined using a freeze‐drying microscope or deduced from the glass transition temperature (Tg). Depending on the sample's composition, Tc can vary from Tg by 2°C to 20°C (Kumbhar et al. 2022). As cautious estimates of the collapse temperature may result in longer operating times, it is only helpful in severe cases where the sample is hard to freeze‐dry.

At least 30% more time is needed for secondary drying than sublimation, making it a slower step in freeze‐drying. By desorption, unfrozen or bound water can be successfully removed, even though it can be done at a temperature lower than the glass transition temperature of dry particles (Ward and Matejtschuk 2020). The product may collapse and lose quality if the temperature rises before all of the ice is sublimated, making it challenging to tell when primary drying ends and secondary drying starts (Pisano 2022). Many techniques have been proposed to determine the primary drying endpoint: these include the use of the Pirani pressure gauge, condenser pressure, dew point monitor, tunable diode laser absorption spectroscopy (TDLAS), gas plasma spectroscopy, and thermocouple (TC) (Bhatta et al. 2020).

## 6 Freeze‐ Drying in Nutraceutical Applications and Food Ingredients

### 6.1 Food Flavors

The process of encapsulating tastes in a continuous matrix produces solid matrices with distributed flavors that are shielded against oxidation and loss. In this procedure, an emulsion of the taste in an aqueous phase made of components soluble in water is prepared, frozen, and then frozen again (Gómez‐Gaete et al. 2024). As a result, the flavor gets stuck in a glassy matrix as tiny droplets. Higher degrees of flavor retention can be achieved by spray‐drying encapsulation using techniques like reduced particle sizes and stable emulsions. The exact encapsulation materials and emulsion properties utilized for flavor spray‐drying are also effective for freeze‐drying (Saifullah et al. 2019). For encapsulated flavors, freeze drying is a drying method that provides excellent product quality and stability. According to studies, it works best for producing dried powders with the desired qualities compared to spray‐drying, tray drying, and drum drying. Gum Arabic, as the best wall material, oxidizes more slowly than hot air dried goods, making it the most effective for encasing menthyl linoleate (Chhabra et al. 2023).

However, there is a dearth of research on taste encapsulation using freeze‐drying. Research by Kaushik and Roos (2007) found that the largest amount of limonene was preserved in emulsions homogenized at an optimal pressure of 100 MPa. Gum Arabic was the best wall material because of its superior drying properties and high retention rates. When gum Arabic and α‐cyclodextrin were utilized as encapsulating materials, freeze‐dried pear fragrance emulsions had high stability and reasonable retention rates (Sagiri et al. 2016). The yield of encapsulated d‐limonene products rose with the wall material concentration, from 5% to 10%. With a percentage of 93.35%, the product, including the hydrophobically modified starch wall material, had the highest. Kaasgaard and Keller (2010) investigated the emulsion qualities of a combination of an oppositely charged polysaccharide (chitosan) and a negatively charged small‐molecule emulsifier (citric acid ester) using freeze‐drying of flavor oil (R‐carvone) emulsions.

### 6.2 Probiotics

The most popular technique for keeping microorganisms, especially probiotics, alive is freeze‐drying; the main reason that culture collections worldwide utilize it. Encapsulating probiotics involves freezing the bacterium at a low temperature in the presence of a carrier material and then freeze‐drying (Liu et al. 2019). Cryoprotectants, such as proteins, carbohydrates, and polymers, shield the microbe from stress during the drying process and storage conditions (Rockinger et al. 2021). The technique's viability and stability are vital. These substances combine to generate an amorphous, glassy state that reduces molecular movement by creating enough viscosity close to the probiotic cells. For instance, trehalose and other compounds having greater total glycerol (Tgs) are better at keeping biological components from becoming denatured during drying and storage. Moreover, hydrogen bonds between sugars like trehalose and biological structures help cryoprotectants, especially those that protect against freezing and dehydration stress, stabilize biological structures (Wang et al. 2022).

Fermentable meals, known as prebiotics, encourage the development and activity of good bacteria in the host's colon. Synbiotics, made up of probiotics and prebiotics, benefit health when taken in moderation (Simon et al. 2021). Probiotic microencapsulation has given rise to synbiotics, wherein the probiotic‐hydrocolloid precursor is supplemented with 1%–2% insoluble starch grains to preserve microbial viability. However, according to a study, co‐encapsulating prebiotics (resistant starch corns) lessened the beneficial effects of the probiotics. Lower survival rates and diminished physical barrier function could arise from RS‐corn present, disrupting the protein matrix's homogeneity. Prebiotics and wall materials must be carefully chosen when co‐encapsulating probiotics to prevent adverse interactions and enhance the protection of probiotics (Kvakova et al. 2021).

The annealing process aims to create pores and channels that facilitate vapor escape during drying by gradually raising and lowering the temperature. By decreasing the freezing rate and increasing the size of the ice crystals, this treatment speeds up primary drying and improves the look of freeze‐dried goods (Oyinloye and Yoon 2020). Large ice crystal production, however, might weaken cell membranes and decrease cell viability. Rapid freezing is advised for freeze‐drying probiotics. High viability (> 60%) for spray freeze‐drying *Lactobacillus paracasei* utilizing maltodextrin and trehalose wall materials was shown in a study by Semyonov et al. (2010). The viability of the bacteria was unaffected by the spraying stage, and the freezing step's intense osmotic pressures due to enhanced trehalose concentrations helped maintain cell viability (Misra et al. 2022). The reduced molecular weight of maltodextrin further enhanced bacteria's survival during the freezing and drying processes.

### 6.3 Enzymes

Enzyme encapsulation is essential to preserving their stability and enabling the repurposing of costly enzymes (Maghraby et al. 2023). Enzymes can become deactivated and unstable during freeze‐drying encapsulation due to many factors, including ice crystal formation, dehydration, and low‐temperature stress. Enzymes can also be shielded by cryoprotectants added to probiotics (Tyutkov et al. 2022). For instance, under ideal circumstances, coating agents such as Arabic gum, maltodextrin, and calcium chloride have efficiently protected freeze‐drying serine protease with an encapsulation yield of 92%. Serine protease activity is enhanced by combining gum Arabic and calcium chloride, with gum Arabic having the greatest effect. The core‐to‐wall ratio highly influences the retention and release of the core during encapsulation. Hashmi et al. (2021) achieved 70% encapsulation efficiency and 80% retention of enzyme activity by encasing flavourzyme in an alginate matrix, gelling it in chitosan containing calcium chloride, and freeze‐drying it. The cheese business may find use for this encapsulated enzyme in creating flavors.

According to a study reported by Ma et al. (2023), trehalose was necessary to protect invertases throughout freezing, drying, and heat treatment. However, it did not avoid damage to enzyme functioning during bead production. Some encapsulation methods related to freeze‐drying are from the literature review in Table 3. Enzyme activity losses during bead formation were shown to be prevented by pectin or β‐cyclodextrin, but these substances had no positive effects on the preservation of enzyme activity during drying or heat treatment. The drying had an impact on the beads' macroscopic and microscopic structure, resulting in lower enzyme release rates from freeze‐dried beads than from vacuum‐dried ones (Shomal et al. 2024). To encapsulate fluoroluciferase by freeze‐drying, Liu et al. (2011) used a colloidal suspension of a chitosan and xanthan gum blend as the wall material. Because montmorillonite nanoclay had a considerable effect on the structural alteration of the polymeric network structures, its addition dramatically slowed the enzyme release rate. The pH of the buffer impacted both the encapsulation stability and the release rate of the enzyme (Weng et al. 2023).

**TABLE 3:** Liposomal lyophilization (freeze‐drying) in food applications.

Liposome composition	Encapsulated compound	Lyophilization parameters	Cryoprotectant/Stabilizer	Encapsulation efficiency (%)	Particle size (nm)	Stability/Retention	Food application	References
Soy phosphatidylcholine	Enzyme (e.g., lactase)	−40°C freezing, 0.1 mbar vacuum	Sucrose	80	200	92% after 6 months	Cheese processing	Alemán et al. (2019)
Phosphatidylcholine, cholesterol	Vitamin D	−50°C freezing, 0.05 mbar vacuum	Trehalose	75	190	90% after 5 months	Fortified beverages	Farzaneh et al. (2018)
Egg lecithin	Antioxidant peptide	−45°C freezing, 0.08 mbar vacuum	Mannitol	83	220	87% after 4 months	Meat preservation	Budai et al. (2013)
DSPC:Chol +5% Scurose	Ovaalbumin (OVA)	−50°C freezing, 0.05 mbar vacuum	Sucrose	32	250	70 after 3 months	Tropical use	Leung et al. (2022)
DOPE/CH/DSPE‐mPEG + Sucrose (8:1 sugar: lipidmass ratio)	Doxorubicin (DOX)	−40°C freezing, 0.03 mbar vacuum	Sucrose	72	180	85 after 6 months	Pharmacutical application	Aloss and Hamar (2023)

## 7 Applications in the Food Industry

**FIGURE 5:**
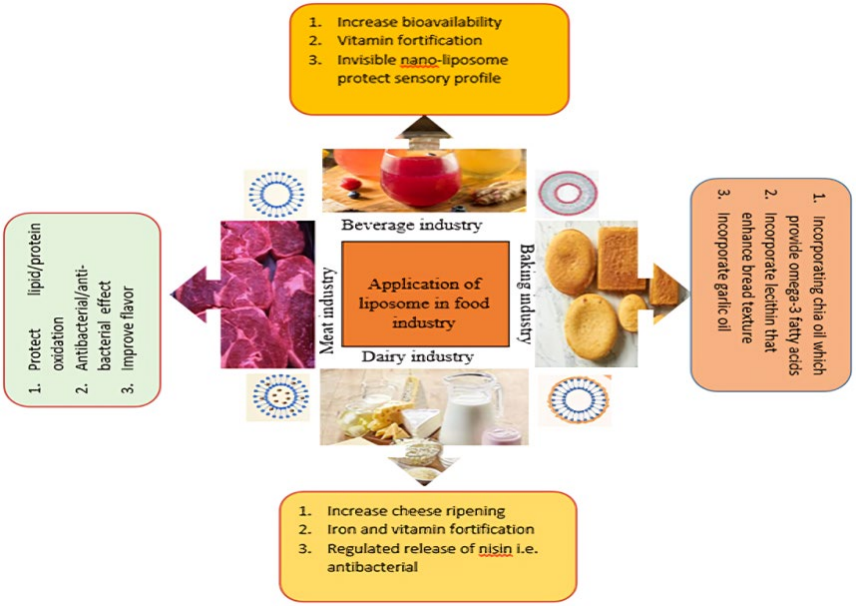
Food application of liposomes in different food industries.

The culinary, pharmaceutical, agricultural, and bioprocessing industries have shown significant interest in liposomes because of their advantageous properties and their higher solubility (Figure 5).

### 7.1 Dairy Industry

The use of liposomal applications in dairy products has been thoroughly investigated in terms of improved antimicrobial peptide delivery and food component stability against degradation (Hudiyanti et al. 2018). Iron deficiency is a well‐recognized nutritional concern that affects people worldwide today. It is mainly caused by a lack of bioavailability, dietary intake, or an inadequate amount of iron, one of the essential components in blood formation. This phenomenon should not be ignored since it may result in anemia, a condition with insufficient iron in the blood, causing the hemoglobin level to drop below normal (Shubham et al. 2020).

Studies on using nanoliposomes containing the enzyme proteinase in cheese primarily focus on cheddar cheese. Timilsena et al. (2020) suggested using fungal or bacterial proteinases that have been encapsulated to speed up the ripening process of cheddar cheese without causing flavor or texture issues. Liposome enzymes shortened the time needed for cheese to ripen by up to 50% (Mohammadi et al. 2021). In contrast to traditional methods, which require a considerable time of more than 1 year to maximize the acceptability, the encapsulated sources can reduce the ripening period of cheese and cheddar, hence increasing the economic profitability of the manufacturer (Oštarić et al. 2022). Applying liposome‐encapsulated enzymes increases the amount of concentrated enzymes involved in flavor development in the curd compared to the dispersion of unbound enzymes in the whole‐milk mixture (Mohammadi et al. 2021).

Apart from proteinase, starter cultures play a significant role in developing the enhanced sensory characteristics of various cheese types. Including lactobacillus cultures during the cheese‐making process enhances textural attributes and aromatic compound production throughout the ripening period (Khattab et al. 2019). Traditional techniques of encapsulating enzymes to speed up cheese ripening have an advantage over chemically or detergent‐induced liposomes in cheese manufacturing because of their lower encapsulating productivity (Jahadi et al. 2015). An enzyme called Flavourzyme is derived from Aspergillus oryzae. This enzyme is employed throughout the cheese‐making process to quicken the process of maturation, which lessens the cheese's bitter flavor and enhances its flavor (Hashempour‐Baltork and Farshi 2022).

Many *Lactococcus lactis* strains produce nisin, an antimicrobial peptide (304 kDa) of 34 amino acids, including unsaturated and lanthionine residues. Since nisin has been granted the Generally Recognized as Safe (GRAS) designation and is effective in inhibiting the development of gram‐positive bacteria on a broad spectrum, it is utilized as a natural antibacterial in various foods (Faure 2022). It is added straight to the food because of its effectiveness. It has been demonstrated that liposomes are suitable vehicles for the regulated release of nisin within the cheese matrix. Because nisin's membrane was immobilized during its entrapment in liposomes, its stability, availability, and distribution were enhanced. It is preferred that the antibacterial activity of nisin be used to preserve cheese for both short‐ and long‐term periods by releasing encapsulated nisin and desorbing membrane‐immobilized nisin, respectively (Yousefi et al. 2023).

Polyunsaturated fatty acids (PUFA) aid in maintaining healthy brain and retinal function and offer protection against cancer, heart disease, and autoimmune disorders. Therefore, we must include the recommended quantity of PUFA in our diet. Fish, in particular, are an abundant marine source of PUFA (Ghorbanzade et al. 2017). Food that has been supplemented with fish oil or PUFA‐incorporated gives off a noticeable fish oil flavor. Fish oil may be enclosed using liposome technology to enhance its sensory qualities. Fish oil that has been nanoencapsulated using nanoliposome techniques was created by Ghorbanzade et al. (2017) and then added to yogurt for fortification. Overall, the findings showed that the sensory parameters of yogurt containing nanoencapsulated fish oil were more similar to those of yogurt containing unencapsulated fish oil.

### 7.2 Baking Industry

A typical cereal food that consumers like most everywhere is bread. Since bran and germ are removed to create refined flour, used to make most bread, it is deficient in vital nutrients like vitamins, minerals, and antioxidants (Gómez et al. 2020). When making bread, the dough typically goes through a heating process in addition to grinding and refinement. While baking is necessary for the development of the dietary and structural characteristics of bread and its digestibility and palatability, it also causes several bioactive components to be reduced or even destroyed (Awulachew 2020). It is claimed that supplemented bread, achieved by adding biologically active compounds or adding a missing component into the formulation, is better able to satisfy the consumer's health and nutrition demands than regular bread. Although the direct addition of bioactive chemicals has also been tried, this application may be limited due to unfavorable taste and flavor.

Furthermore, bread fortification is restricted by high volatility, low bioavailability, low bio accessibility, and heat instability (Di Cairano et al. 2022). Bioactive substances resolve this issue, but before being used for food enrichment or fortification, they might be encapsulated in protective matrices to overcome these and other challenges. Encapsulation is the process of encasing materials in their solid, liquid, or gaseous phases in a covering or putting them in a matrix of different‐sized particles (Abbas et al. 2022). Numerous researchers have utilized encapsulation technology to make functional bread containing encapsulated probiotics, enzymes, vitamins, polyphenols, and omega‐3 fatty acids (Rousta et al. 2021). Several scientists are working on creating bread using bioactive substances that are encapsulated. Minerals and vitamins are vital micronutrients that support human growth, development, and metabolism (Gharibzahedi and Jafari 2017). Of all the vitamins, vitamin D deficiency is the most common globally. One of the most important forms of vitamin D for controlling calcium and phosphorus homeostasis is calcitriol, a fat‐soluble precursor of vitamin D. Food fortification techniques can combat vitamin D deficiency. But because vitamin D is highly susceptible to oxidation when exposed to extreme temperatures, daylight, humidity, or oxygen, it can be difficult to include it in food (Dominguez et al. 2021). Zhu et al. (2021) investigated the microencapsulation of vitamin D with egg white and its use for bread enrichment to get around this problem. The microcapsules have a long shelf life and antimicrobial qualities. The encapsulated vitamins were shielded from deterioration under cooking, simulated storage, heating, and UV irradiation. According to the in vitro digestion study, functional vitamin D has the potential to be produced in the gastrointestinal tract, increasing its availability by almost 2 times when compared to free vitamin D. The vitamin D microcapsules were highly stable and kept their microstructures after being added to common food items.

In a study conducted in 2018, González and colleagues (2018) investigated the enhancement of bread by incorporating encapsulated chia oil, with soy protein serving as the coating substance. They evaluated the hydroperoxide values during storage for up to 14 days. Including encapsulated chia oil did not affect bread's technological characteristics and effectively hindered the formation of hydroperoxide radicals. Sixty percent of the daily required consumption of omega‐3 fatty acids can be obtained from a ration of bread containing encapsulated oil. These results could be explained by the emulsifying capabilities of lecithin's surface and other liposomal system elements that enhance dough stability, gas retention, and bread volume. Nano‐liposomes likely benefit from extending the shelf life of bakery items. Because lecithin and glycerol emulsifiers are included in the liposome structure, nano‐liposomal samples have a lower hardness. Mono and di‐glyceride emulsions significantly enhance the softness and maintain the freshness of the bread's crumb. Essentially, these compounds impact specific components of the flour, mainly its starch.

Ojagh and Hasani (2018) observed that bread fortified with liposome‐encapsulated fish oil exhibited a 5% increase in volume upon adding microcapsules. Technological and sensory assessments revealed that bread enriched with nano‐liposomal fish oil displayed superior loaf volume compared to other samples. Incorporating nano‐liposomal fish oil did not adversely impact the bread's textural quality or sensory appeal. Therefore, the utilization of nano‐liposomal fish oil as a beneficial additive to enhance the nutritional content of bread is recommended, along with its potential application for the production of other bakery items. Major polyunsaturated fatty acids, including omega‐3 (EPA and DHA, or eicosapentaenoic acid), omega‐6, and oleic acid, are found in walnut oil (WO). These fatty acids have many health‐promoting properties, including pro‐inflammatory responses, cancer prevention, and protection against degenerative diseases like cardiovascular disease (Adelakun et al. 2019). Despite the advantages, omega‐rich oils are highly vulnerable to oxidation, presenting a significant challenge regarding food products' applicability, storage acceptability, and nutritional properties. Encapsulating omega‐rich oils within a protein‐polysaccharide matrix has been widely used for food fortification. Cream‐filled sandwich cookies fortified with polyunsaturated fatty acids (PUFAs) demonstrate no adverse effects on the organoleptic properties of the base product (Borneo et al. 2007; Rahim, Regenstein, et al. 2024). Because the emulsion remains stable in various food matrices, encapsulating WO in a protein‐polysaccharide matrix can prevent fatty acid oxidation and be widely used by rising food businesses to protect omega fatty acids (Bahrami et al. 2020).

In general, changes to the sensory aspects of bread fortified with microencapsulated bioactive substances occur concurrently with modifications to the technical and rheological properties of the bread. The crumb color, scent, taste, and general acceptability of the bread samples were all improved by adding fish oil nano‐liposomal capsules, and these results were comparable to those of the control sample (Ojagh and Hasani 2018). Since ancient times, garlic ( *Allium sativum* ), one of the earliest medicinal herbs utilized in various civilizations, has been used to cure and prevent various illnesses (Rahim, Yasmin, et al. 2022). The main qualitative factor that affects bread texture is hardness. Bread products should have low hardness levels. Bread fortified with garlic oil and microencapsulated received the lowest possible hardness score, which suits the bread's quality. The hydrophilic properties of alginate microcapsules diminish hardness, potentially through moisture retention. The crumb's softness could result from the release of garlic oil, which replenishes over time during storage. The presence of free fat on the surface contributes to decreased hardness values. Enhancement in sensory attributes was also noted (Narsaiah et al. 2019).

### 7.3 Meat Industry

Meats are a dense matrix with good structural and viscoelastic qualities. They are also highly concentrated in water, vitamins, protein, and unsaturated or saturated fat. They can be further processed to make ready‐to‐eat meals like burgers, dumplings, sausage, and bacon. But during preservation and processing, meats frequently suffer from oxidation of the lipids and proteins. This can lead to the creation of hydrogen peroxide, volatile chemicals, and derivatives of genotoxic amino acids, which can negatively impact the digestibility, availability, and sensory appeal of meat products (Huang et al. 2022). Propyl gallate (PG), tert‐butylhydroquinone (TBHQ), butylated hydroxyanisole (BHA), and butylated hydroxytoluene (BHT) are examples of synthetic compounds that are commonly used to preserve meat quality. However, consumers prefer natural antioxidants because they are concerned about the possible toxicological effects of synthetic ones (Stoia and Oancea 2022).

Due to the severe environments, naturally active chemicals tend to volatilize easily and degrade quickly, resulting in a reduced action period and diminished effect. Sustained‐release systems can produce a prolonged effect through the gradual release of active substances over a protracted period. To limit the essential oil's strong aroma and lessen the silver nanoparticles' toxicity, researchers encapsulated laurel essential oil in liposomes (Nair et al. 2022). Bioavailability is the percentage of active components the body absorbs into the blood flow after consuming them (Chen et al. 2022). The poorly water‐soluble compounds would be transferred and solubilized into the hydrophobic core of the liposomes after hydrolysis and reconstruction to form mixed micelles of phosphate lipid, bile salt, and fatty acid chains. This would increase the solubilization of the hydrophobic compound under physiological conditions (Liu et al. 2018). Iron pyrophosphate has been shown in studies to increase the bioavailability of iron when it is encapsulated in liposomes and incorporated into pork pate as a fortifier to help avoid human iron insufficiency (Huang et al. 2022).

Oxidation and foodborne germs are the main variables that impact the condition of beef products before, during, and after storage (Pateiro, Gómez, et al. 2021). Antibacterial and antioxidant compounds with functional properties are being studied to prolong the shelf life of meat and keep it fresh. Meat preservation uses liposomes, a kind of active food carrier. Lipid oxidation causes meat to lose its nutritional value and change in flavor and color, which poses health risks and causes financial losses. The interactions of free radicals between oxygen and fatty acids are incredibly intricate. The same free radical chain reaction that causes lipids also causes protein oxidation in beef (Nawaz et al. 2022). Proteins, fats, water, and vitamins are all found in high concentrations in meat products and are essential for developing and reproducing microorganisms. Antimicrobial and antioxidant agents, including peptides, bacteriophages, essential oils, and bioactive compounds, have been loaded into liposomes in the meat industry to defend against hazardous microbial material and oxidation of lipids and proteins (Huang et al. 2022).

Essential oils can function as active components in food packaging and as preservatives. Notwithstanding all of these uses, less stability, heat sensitivity, and the strong smell of essential oils are the greatest limitations to their efficient use (Rahim, Imran, et al. 2023; Eranda et al. 2024). Liposome technology is the best solution for this problem. Nutmeg oil is the main functional component of nutmeg, which is volatile and has antibacterial and antifungal properties. However, losses were observed even after 5 days of fortification due to its volatile nature. Nutmeg oil was incorporated in liposome technology to overcome this issue for its better and long‐term effect (Huang et al. 2022). Another study investigated the effect of nutmeg oil in encapsulated form, which showed a better antimicrobial effect in *L. monocytogenes* in dumplings (Zhu et al. 2020). Similarly, thyme oil‐encapsulated liposomes, as opposed to free thyme oil, might significantly enhance the bacteriostatic activities against *S. enteritidis* in chickens without affecting their health or having an undesirable sensory effect (Kaur and Kaur 2021).

### 7.4 Beverage Industry

The ability of nano‐liposomes to typically elude human perception is one of the main benefits of their use in the food industry. This allows for adding bioactives (like omega fatty acids derived from fish) to food and drink without significantly altering the sensory qualities of the original product. Nano‐liposomes are undetectable to the unaided eye due to their nanometric size. If they are kept below 80 nm in diameter (and not at exceptionally high concentrations or particle refractive index not significantly different from the solution), they hardly reflect visible light and maintain transparency. These covert vesicles come in handy when adding hydrophobic nutrients or flavorings to transparent beverages that do not taste or smell well (Singh et al. 2023).

Provitamin A, or beta‐carotene, protects against lung cancer and cardiovascular disease and prevents vitamin A insufficiency (Didier et al. 2023). Because beta‐carotene is hydrophobic, it has poor chemical stability and low bioavailability in liquid food preparations. Liposome techniques incorporating spray‐dried phospholipid particles may increase beta‐carotene solubility (Toniazzo et al. 2014) and active loading methods (Hudiyanti et al. 2018) components for sesame‐based liposomal substances. The microencapsulated molecules' physical, chemical, and biological characteristics impact their absorption at the intended delivery locations and prevent them from entirely surviving the gastrointestinal system. The nano‐liposome techniques host substances with low molecular weight compounds, such as bioactive substances, proteins, peptides, and nucleic acids, that gradually release their biologically active components for a prolonged time to the target region in both passive and active targeting delivery pathways. This was done to improve the solubility and absorption effectiveness due to the presence of more surface area in the nanoliposome. This lowers the amount of the substances, reducing both the toxicity and adverse effects. In contrast, sick or damaged cells are affected; as a result, healthy cells are unaffected (Singh et al. 2023).

Orange juice is pasteurized after being added to liposomes based on soy phosphatidylcholine, which were investigated as vitamin transporters. These technologies made it possible to include vitamins C and E. Instead of using lipid bilayer stabilizers, calcium stearate and stearic acid were added to liposomes. It is crucial to remember that adding vitamins and liposomal formulations to orange juice did not alter its organoleptic properties, and following pasteurization and storage, it demonstrated microbiological stability (Marsanasco et al. 2011).

Calcium stearate has additional nutritional significance because it contains calcium ions and essential fatty acids. The high amount of polyphenols in tea makes them reactive to light and oxygen. This compound, found in green tea, is widely used in food products and has reputable biological and pharmacological actions. It has been shown to have antioxidant properties by accepting free radicals, chelating metal ions, donating hydrogen atoms, inhibiting oxidation chain reactions, and lowering blood lipid levels (Mangunsong et al. 2023). The tea polyphenol liposomal product was created using a thin film ultrasonic dispersion technique, increasing the component's bioavailability (Jara‐Quijada et al. 2024).

## 8 Liposomal Technology in Nutraceuticals

Natural chemical substances with therapeutic properties are known as “nutraceuticals,” meaning they have both medicinal and nutritional value. Building on the effective application of liposomes in the biomedical and pharmaceutical industries, scientists are now using nano‐liposomes for the encapsulation and controlled release of nutraceuticals and functional food ingredients. These include antioxidants, proteins, polysaccharides, enzymes, vitamins, flavors, preservatives, and essential oils (Pateiro, Munekata, et al. 2021).

Appropriate dietary supplemental carriers are required to deliver the compounds to a particular target location of the cells. These carriers must also protect the compounds and improve their bioavailability. Manufacturing appropriate encapsulated nutraceutical materials with nutraceutical delivery and carrier qualities faces this problem. Liposomes can significantly alter nano carrier products by using a customized product‐enabling system. These products can be applied to the biological site inside (such as when antibodies are integrated) or outside (depending on particle or cell size) of a human body in a variety of target‐oriented dietary supplements (Subramani and Ganapathyswamy 2020).

*Curcuma longa* (turmeric) rhizomes contain a naturally occurring polyphenol called curcumin. According to reports, curcumin demonstrates anti‐inflammatory, anti‐tumor, antioxidant, and chemopreventive properties (Alabdali et al. 2021). It is a hydrophobic molecule that is poorly absorbed in the intestine. After oral administration, it undergoes biotransformation, leading to its elimination through bile secretion. Liposomal encapsulation is recommended for delivery (Stohs et al. 2020). Nanoliposome techniques use substances like low molecular weight compounds, bioactive compounds, proteins, peptides, and nucleic acids to improve solubility and absorption in both passive and active targeting pathways. This reduces substance quantity, toxicity, and side effects, ensuring healthy cells are not affected while unhealthy or damaged cells are affected (Wagle et al. 2020).

In human systems, iron is essential for several physiological processes, including erythropoiesis, metabolic oxidation, and immune function in cells. Using liposome technology to encapsulate micronized iron offers new opportunities for increasing iron consumption through oral therapy. It also has a specific cell delivery mechanism and is associated with the least exposure to different stomach contents and digestive enzymes and the least amount of food component interface activity (Maladkar et al. 2020). Using two different methods, Cantor et al. (2019) investigated the antibiotic activity of peptide‐based nanoliposomes against *Escherichia coli* and *Listeria monocytogenes* . Antimicrobial peptides were changed structurally in one process, and the modified peptides were then nano‐vesiculated into polymer‐coated liposomes in a different method. The findings demonstrated that the degree of hydrophilic alteration in the peptide causes distinct amphipathicity traits, which in turn cause distinct physicochemical behaviors in microorganisms.

## 9 Liposome Microencapsulation in the Agriculture Sector

Many developing nations rely heavily on agriculture to boost their national economies. Hence, it is vital to establish quickly expanding and sustainable agricultural systems. This could be due to several factors, including the population's steady and quick rise, climate change, and the ongoing depletion of natural resources, in addition to the cumulatively harmful effects of using conventional chemical pesticides and fertilizers on the environment (Devenish et al. 2023). In the past, nanotechnology has been applied to various disciplines, including medicine, chemistry, pharmaceutical research, and diagnosis, to mention a few. At the vanguard of nanotechnology, nanoparticles (NPs) are used in many consumer items because of their enormous potential compared to their bulk counterparts. As a result, the food and agriculture industries have taken notice of these novel materials' distinctive and developing qualities (Mittal et al. 2020).

The innovative uses of nanotechnology in agriculture impact agricultural productivity by using NPs as nanocarriers to transfer advantageous DNA, improve genetic features, and improve seed breeding. Additionally, giving the plants the nano nutrients and nano fertilizers they need to decrease nutrient waste in the soil, enhance soil quality, and shield the soil microbiome from any harm caused by nano residues (Predoi et al. 2020). Usually, medicines, fertilizers, and insecticides that may be effectively washed off are sprayed. Moreover, these fertilizers and insecticides have very high production costs, which must be controlled. Using NMs in agriculture seeks to lower product amounts for plant protection while reducing nutrient losses to boost yields. Additionally, by using nanopesticides, nanoherbicides, and nanofungicides, nanotechnology can improve plant protection (Usman et al. 2020), whereas it can increase a plant's ability to withstand adverse environmental factors like salinity, drought, and UV‐B (Vermeulen et al. 2012; Khalid et al. 2022).

Agronomic approaches that biofortify edible crops have been considered a substitute to lessen the deficiency of Se in people resulting from soil deficiency in selenium (Hossain et al. 2021). Se‐biofortification of wheat has been chosen due to its significance in society's customary diet. Most people frequently eat it in significant quantities, and it is an affordable diet that can be enhanced with additional nutrients like selenium. Through foliar spray, liposomes have been utilized as nanocarriers for biofortifying wheat plants with selenium (Se) (Viltres‐Portales et al. 2024). Liposome nanocarriers can resolve the issues of pesticide instability, quick degradation, and short half‐life. Yeast cells are prototypical fungal targets, and their growth was effectively reduced by adding sterosomes loaded with cymoxanil to the culture media (Zhang et al. 2021). Moreover, NPs can be utilized to create nanosensors, which can be used in precision farming and smart agriculture to track and manage plant health, development circumstances, and soil characteristics (Shawon et al. 2020).

## 10 Conclusions

Liposomal microencapsulation stands as a promising technique for the food industry, offering versatile solutions to protect, deliver, and enhance the functionality of bioactive compounds, enzymes, and other food ingredients. This review has highlighted the fundamental aspects of liposomes, including their structure, properties, and formulation methods, with a specific focus on their applications in food systems. The ability of liposomes to encapsulate both hydrophilic and hydrophobic substances, coupled with their biocompatibility and potential for controlled release, makes them a superior alternative to traditional encapsulation methods. The impact of spray‐drying and freeze‐drying on liposome stability and functionality has been explicitly evaluated through detailed comparisons of processing parameters and their outcomes. Key effects on particle size distribution, membrane integrity, and encapsulation efficiency have been discussed, with supporting data from recent studies. For instance, the inclusion of cryoprotectants like trehalose during freeze‐drying significantly reduces vesicle fusion and leakage. Similarly, optimized inlet temperatures and carrier materials in spray‐drying have been shown to preserve liposomal structure and active compound stability. These evaluations provide practical insights into selecting appropriate techniques for food‐grade liposomal formulations. This review discusses the importance of optimizing liposome processes and selecting appropriate cryoprotectants for effective delivery of encapsulated compounds. It highlights the diversity of liposome formulations and their adaptability for food applications. Challenges include improving scalability, stability, safety, and regulatory compliance. Future research should focus on novel formulations.

## Author Contributions

M.A.R. and H.M.J. wrote the original draft. F.C. and M.F.R. conceptualization and did funding acquisition. M.F.R. and S.A. helped in writing this manuscript. While H.M.J., E.Z. and S.A. helped with the software, M.F.R., H.A.Z., R.C.‐M. and M.A.R. improved and supported the writing, review and editing. F.A.‐A. resources. All authors have read and agreed to the published version of the manuscript.

## Conflicts of Interest

The authors declare no conflicts of interest.

## Data Availability

Data is contained within the article.
